# On Small-Pox after Vaccination

**Published:** 1817-01

**Authors:** Thomas Harrison


*For the London Medical and Physical Journal.*

### *On Small-Pox after Vaccination;* by THOMAS HARRISON, Esq.

EARLY in last spring, a report prevailed in Kendal that the Small-pox had appeared in Ulverston; and that many persons had been infected with them who had been previously vaccinated, and thought secure. In consequence of this rumour, I was induced, in May last, to request my friend Mr. Redhead, a respectable and intelligent practitioner in that town, to procure all the information on the subject he could, respecting not only the cases which had fallen under his own observation, but also those which had occurred in the practice of the other medical men in the place. In order to obtain a correct statement of the facts relating to this report, I addressed the following queries to him:—

When were the small-pox introduced into Ulverston?

What number of persons have had the small-pox, after having gone through the cow-pox?

By whom were they vaccinated?

At what day of the disease was the cow-pox matter usually taken?

Have

Have those persons who have had the small-pox after vaccination previously had the chicken-pox?

At what age where they vaccinated?

At what age where they infected with the small-pox?

Were they of a favourable or unfavourable kind?

Did any of the patients die of the disease?

Have any persons been inoculated with the small-pox who had previously gone through the cow-pox, and what was the result?

Have the small-pox infected many persons who have never been vaccinated; and have they been mild or severe?

In the beginning of July, I received the following letter, together with the cases which had occurred.

---

DEAR SIR, *Ulverston; 3d July,* 1816.

THE small-pox were brought to Ulverston from Wigan, by the wife of a nailer, who, with her child, had slept in a house where the family had just recovered from them, in the latter end of January, or beginning of February. She immediately returned to Ulverston, and the eruption appeared on the child about ten days afterwards, when it was carried about by the mother, and much exposed in different parts of the town. They soon removed from this place; and I believe the child died between this place and Kendal.

A young woman of the name of Wilkinson, who was in the habit of going to the house where the child lodged, soon became infected with the small-pox, and died on the eleventh day of the eruption: my first visit to her was on the 20th February, and she died on the 22d. Her sister began soon afterwards, and had the complaint favourably.—None of these persons had been vaccinated.

With respect to the complaint which took place after vaccination, the first case which I saw was that of John Turner's child; and I was inclined to believe that it was not small-pox, until her sister began, when I was obliged to consider them as cases of small-pox after vaccination, for they had both of them previously had the chicken-pox. Robert Woodhouse's daughter was the next case, and she had previously gone through the chicken-pox, and had been blind in them.

The usual time of taking matter from the cow-pock is the eighth day,—I believe seldom, although sometimes, either earlier or later; and, as far as I have observed, the cases of cow-pox, under the care of the other medical practitioners in this town, have assumed the same appearances

 as as those in my own practice: and vaccine lymph, procured from London, or any other place, produces still the same appearances, or nearly so. I have also observed, and find it to be the case with the other medical men, that, in not a few instances, the pustule is smaller than in general, and in other instances later, and consequently smaller; and that, with these appearances, the inflammation is generally less, and sooner subsides.

In many of the cases which I have sent you, matter was taken from the patients during vaccination; consequently they were not disputed cases of cow-pox. Until this appearance of small-pox after vaccination, no experiments had been made, for a long time, to prove the security of persons from small-pox who had been vaccinated; but soon after the cow-pox was first introduced into this part of the country, many persons, after having been vaccinated, were inoculated with variolous matter; and others were exposed to the contagion of small-pox, by being carried to the houses of persons labouring under that complaint in its full virulence,—yet not one of these were infected with the small-pox. These were considered as convincing proofs of the preventive power of the cow-pox.

I am, dear sir, your's, &c.

M. Redhead.

---

The four following cases of natural small-pox are here introduced to point out the source of infection.

1. A nailer's child. Was infected at Wigan, in Lancashire, where it slept in a house in which a family was just recovering from the small-pox: during its sickness, was carried by the mother round the town of Ulverston.

2. —— Wilkinson, a young woman, who was infected by visiting the nailer's child, and died on the eleventh day of the disease.

3. Wm. Barber's daughter, 12 years of age. The pustules were distinct, and she recovered well.

4. Geo. Garnett, 40 years of age. Had a very full crop of distinct pustules; but he recovered with difficulty. In the same house were four children, who had been previously vaccinated, and two who were vaccinated after he began; but none of these were infected with small-pox.

*The*

### *The following are Cases of Natural Small-pox after Vaccination.*

**Those marked thus * had vaccine lymph taken from the arm during vaccination.—Those marked thus † had previously gone through the chicken-pox.**

| Name. | Age when Vaccinated. | By whom. | Age when infected with Small-pox | Nature of the Small-pox. | Observations. |
|---|---|---|---|---|---|
| 1 Robert Jones's two children | *infants | Mr. Redhead | 10 & 8 | Small horny pox, which continued out only 5 or 6 days—were not seen by a medical man. | One child had considerable fever, during 4 days previous to the eruption. There had been a regular communication between Geo. Garnet and the household of this family. |
| 2 Elizabeth James | infant | Mr. Harrison | 12 | † Very feverish; pustules distinct—a well-marked case of small-pox. | Had been much exposed to Wm. Barber's daughter in the small-pox. Matter was taken from her on the 7th day, to inoculate the small-pox. |
| 3 Joseph James | do. | Mr. Redhead | 15 | Very feverish, and thought dangerously ill for a few days—not so full as Elizabeth. | Was infected from her. An apprentice, exposed in the same manner, who had been previously vaccinated, did not receive the infection of small-pox. |
| 4 William James | do. | Mr. Carter | 8 | Had them milder than the two former; continued out a few days. | Ditto from Elizabeth. |
| 5 William Parker | 4 | Mr. Close, Dalton | 18 | Delirious two days before the eruption appeared; pustules numerous, and continued out 7 or 8 days. | Do. from Eliz. James. |
| 6 Elizabeth Fell | 3 | Mr. Briggs | 12 | † Considerable fever previous to the eruption, which was of the distinct kind. | Exposed to Wm. Barber's daughter at the same time with Elizabeth James; and both of them sickened at the same time. |
| 7 Maria Stable | 10 | Mr. Carter | 19 | † Feverish before the eruption, which was of a small horny kind, and soon disappeared. | |
| 8 Mary Stable | infant | Mr. Edmonson, Keswick, | 14 | † Had slight eruption, and was soon well—began about 14 days after her sister Maria. | A brother who had not received the cow-pox infection when vaccinated, was infected with small-pox from Maria, and began at the same time with Mary. He had a full crop of fine distinct small-pox: they were much larger, and continued out longer than his sister's; and he is much marked. Eight children were inoculated from him. Two other children, lately vaccinated, and exposed afterwards to the same contagion, were slightly indisposed, and had a few pustules. |
| 9 Charles Hodge | do. | Mr. Harrison | 9 | Fever, with slight delirium, succeeded by a great number of fine pustules. | |
| 10 John Briscoe | 3 | Mr. Redhead | 16 | † A few small horny pox, which continued out about 5 days. | |
| 11 Thomas Briscoe | infant | do. | 9 | Small horny pox. | |
| 12 Mary A. Briscoe | do. | do. | 6 | Ditto—began to be ill about a fortnight after her brother Thomas. | |

| | | | | | |
|---|---|---|---|---|---|
| 13 Betty Turner | infant | Mr. Redhead | 6 | † Feverish, 3 days with delirium; face full of pustules, and many on her body. | Small horny kind, which disappeared in five or six days. |
| 14 Alice Turner | do. | do. | 12 | † Feverish; not so much indisposed as Betty; had fewer pustules, but larger. | Two other children, which had been previously vaccinated, did not take the small-pox. |
| 15 Rob. Braithwaite's daughter | do. | Mr. Carter | 14 | † Very feverish; had a full crop of small horny pox; face swelled; blind 3 days. | Three other children, who had been vaccinated at the same time, and who never had gone through the chicken-pox, did not take the small-pox. |
| 16 Mr. Rawlinson's son | —— | Mr. Briggs | —— | Much fever; very full of pustules, and is much marked. | |
| 17 Ellen Physaclea | infant | Mr. Redhead | 13 | Very feverish; had large distinct pox; has marks on the face. | Another daughter, vaccinated at the same time by Mr. Redhead, has not been infected with small-pox. |
| 18 Mary Long | 4 | Mr. Carter | 10 | Much fever, with delirium 3 days; succeeded by small horny pox. | The pox disappeared on the 5th day, and left marks on the arms and legs. Three others, previously vaccinated, were not infected. |
| 19 William Baines | *infant | Mr. Redhead | 3 | Had 3 days' illness, succeeded by horny pox, which encrusted in 6 days. | Had been much exposed to a child in small-pox, and became ill about a week after the child was turned the height. |
| 20 William Monkhouse | 2 | Mr. Carter | —— | Not much indisposed; pustules of the horny kind. | Became infected 4 or 5 weeks after vaccination. |
| 21 Betty Clark | infant | do. | 7 | † Feverish 3 days; eruption full, but distinct, and run the regular stages of small-pox. | Four other children, which have been vaccinated, have not taken the small-pox. |
| 22 John Nicholson's daughter | —— | Mr. Harrison's assistant | —— | Slight degree of fever; small number of pox; favourably indisposed. | Four others, previously vaccinated, in the same house, but not yet affected with small-pox. |
| 23 Rob. Woodhouse's daughter | infant | an old woman | 13 | † Very feverish several days; but few pustules, and speedy recovery. | Others in the same family, vaccinated by Mr. Redhead, are not yet affected with small-pox. |
| 24 William Tyson | 2 | Mr. Briggs | 9 | At the height in 7 days. | Mr. Briggs saw him, and had no doubt of the complaint being small-pox. |
| 25 John Kirkby | infant | do. | 11 | Indisposed 2 or 3 days; eruption of the horny kind; disappeared in a few days. | |
| 26 Wm. & Benj. Kirkby | 5 & 3 | Mr. T. Carter | —— | The eruption on William was larger, and continued longer, than the rest. | Pustules of the horny kind. These two children were infected 4 weeks after vaccination. |
| 27 Joseph Kirkby | infant | Mr. Redhead | 5 | Took the infection at Whitehaven; got easily through the complaint. | The small-pox were very fatal at Whitehaven. A sister, not vaccinated, took the small-pox at the same time, but recovered. |
| 28 Sarah Bond | do. | at Liverpool | 12 | † Much fever, with delirium; had many pustules of the horny kind, which soon disappeared. | Supposed to have been infected from Eliz. Fell, as she called to visit Elizabeth. |
| 29 Jane Ellis | do. | Mr. Lodge, Ingleton. | 11 | Had a remarkably full crop; in fact, was one complete cake of incrustation. | Recovered pretty well, but is much marked; was about a month confined. |

| | | | | | |
|---|---|---|---|---|---|
| 30 Betsy Walters | infant | Mr. Harrison | 14 | † Had a small crop of distinct pustules. | Three other children of the same family, vaccinated 4 months ago, and two 7 years ago, have not been infected. |
| 31 Isabella Dixon | do. | do. | 10 | Had a full crop, is marked, but recovered well. | Was exposed to a family in small-pox. |
| 32 Margaret Dixon | do. | Mr. Redhead | 12 | Not very full; pustules perfectly distinct; recovered well. | Began nine days after her sister Isabella was at the height. |
| 33 Betty Garnet | do. | Mr. Carter | 14 | Distinct pustules; was at the height in 8 days, and recovered well. | Was infected from the family of C. Troughaire. |
| 34 Christ. Troughaire's four children | —— | Mrs. Dixon, Mr. Carter, Mr. Briggs | —— | Had them favourably, of the distinct kind; one had a full crop. | Were infected from two children of the same family, which had not been vaccinated, but became infected with the small-pox: one of which died on the eleventh day. Another child, previously vaccinated by Mrs. Dixon, has not been infected with small-pox. The children slept indiscriminately together. |

*The following are Cases of Small-pox after Vaccination, by Inoculation.*

| | | | | | | |
|---|---|---|---|---|---|---|
| 35 Rev. S. Redhead's daughter | infant | Mr. Outhwaite, Bradford, | 5 | † Inoculated: in 8 days became sick and feverish, and had a slight eruption. | Took a little calomel, and was soon well. | |
| 36 Rev. W. Seatle's two children | do. | Mr. Harrison | 14&16 | Ditto: one child feverish, and had trifling eruption of the small horny kind. | The other feverish, but without eruption. | These 8 children were inoculated with variolous matter, taken from J. Stable's son. |
| 37 William Travis's 5 children | —— | not known | —— | Ditto: one child only had slight fever, but no eruption. | | |
| 38 Mr. Rawlinson's two daughters | —— | Mr. Briggs | adults | Ditto: slight fever; eruption of the horny kind. | | |
| 39 J. Stamper's two *children | —— | Mr. Harrison | 12 & 9 | Ditto: slight indisposition; arm much inflamed; very few pustules, which continued out a week. | Another child was not infected, having been previously inoculated with small-pox, and could not be infected. | |
| 40 James Shepherd | 15 mo. | Mr. T. Carter | 18 mo. | Ditto: very feverish; arm much inflamed; pustules not very large. | Inoculated from Elizabeth James, and matter was taken from him. | |
| 41 Thomas Bolton | *infant | Mr. Harrison | 12 yrs. | Ditto: feverish on the 9th and 10th days; considerable inflammation; a few pustules round the part inoculated. | | |

For the purpose of clearly ascertaining that the eruption by which the patients had been affected was the small-pox, I requested Mr. Redhead, in the month of September, to inform me which of the patients had previously gone through the chicken-pox. He very readily made inquiry, and sent me the result, which I have pointed out in the Table of Cases; and at the same time he sent me a few more cases, (which are also placed in the Table,) and the following letter.

---

DEAR SIR, *Ulverston; Oct.* 8, 1816.

I have collected a few more cases of small-pox after vaccination, amongst which is exhibited, in the family of Christopher Troughaire, a striking proof, either of the inefficacy of cow-pock in preventing small-pox in many instances, or of our imperfect acquaintance with the appearances of genuine cow-pox. Troughaire keeps a common garden: he knows not where his children received the infection of small-pox; but it is probable that the contagion was either taken to his house, by persons who were in the habit of going there, or that some of the family (particularly of the children) had been exposed to it, in their daily rounds to dispose of the produce of the garden.

We have repeatedly had the small-pox in Ulverston and the neighbourhood since the introduction of vaccination, yet, until the late attacks, the complaint never extended beyond one or two cases. But I have no doubt, in my mind, as to there having been a great many failures amongst the children vaccinated in Ulverston; yet from what cause I am at a loss to know, but should be very glad to have the matter properly investigated, so as, if possible, to prevent such occurrences in future. It is, however, very extraordinary, that the same disease and appearances have not spread beyond the town of Ulverston; for, although persons infected with small-pox in Ulverston have been removed to the village of Bouth, and to other parts of the country, yet the complaint has not spread. And, about two years ago, many children were inoculated with variolous matter at Pennybridge, a village a few miles from hence; yet no one, previously vaccinated, received the infection from them, except, *probably*, in one instance.

Persons who have had children in the natural small-pox, are convinced that this complaint is so much milder after vaccination, that they would have their children vaccinated, if they were even sure that they must have the small-pox afterwards. I am, dear sir, your's, &c.

M. REDHEAD.

 The

The first question that naturally occurs to the mind, after perusing this account, is, Was the eruptive disease, with which these persons were affected at various distances of time after vaccination, really the small-pox? We find, from Mr. Redhead's first letter, that no such complaint existed in Ulverston previously to the introduction of the small-pox by the nailer's child, in the latter end of January or the beginning of February last; and that soon after that time, several persons were attacked by small-pox, of which some died: and it appears probable that the two first cases, viz. Robert Jones's children, were infected from George Garnet, one of the persons who with difficulty recovered from the small-pox. The 3d (Elizabeth James) and the 7th (Elizabeth Fell) were exposed to the contagion arising from William Barber's daughter, another of those who laboured under the natural small-pox. We observe, also, that a son of Stables, who had not had the cow-pox, took the small-pox from his sister Maria (Case 8), who laboured under that complaint, although she had been previously vaccinated; and the pustules on this boy were so full, distinct, and characteristic of the small-pox, that eight children were inoculated by Mr. Redhead from him. We also may remark, that Maria Stables, and many others who were affected in a similar manner, had previously gone through the chicken-pox. What stronger medical evidence, then, can we expect in proof of this disease being the small-pox? I should have been exceedingly glad to have been convinced, by an attentive perusal of the cases, that they were only the chicken-pox; but candour induces me to confess that I can find no fair grounds for such a conclusion.

The next consideration which occurs is, whether the persons thus affected had been vaccinated with the genuine cow-pock matter; or, in other words, had they been perfectly vaccinated? This is a question of some difficulty, and which admits of no direct proof. But, that the medical men, by whom they had been vaccinated, were of opinion that many of them were secure, is evident, from their having taken matter from the arms of their patients to vaccinate others with: and, that the constitutions of all the patients had been more or less affected by the cow-pox, may be fairly inferred, from the succeeding small-pox being peculiarly mild in all. Had there been but a few patients who had been vaccinated by one person, at the same time; or had they been all vaccinated at the same period, by the respective medical men, with matter obtained from the same source; we might have concluded that the matter had been taken from a spurious vesicle: but, when we perceive that they have been vaccinated by ten different medical men and two matrons, at different periods, and in parts of the country far distant from each other; and that they have been infected with small-pox, either by exposure or direct inoculation at one season, and therefore at various distances of time after vaccination; we cannot but feel our confidence in the preventive power of the cow-pox to be somewhat shaken.

I have subjoined a few cases which happened in London, about two years ago, in the family of Mr. George Rimington, who then lived at No. 29, Ludgate-hill. These cases excited considerable interest amongst some medical men at the time, from one of the children having been vaccinated, at a public institution in London, by Dr. Jenner himself, and, after having been daily inspected, was by him pronounced secure from small-pox.

"Case 1st.—Jane Rimington was inoculated for the cow-pox, at Penrith, in Cumberland, when she was six weeks old, by Dr. Harrison: she had considerable soreness in the axilla; and the Doctor took matter from her to inoculate his patients with. She had the confluent small-pox, at the age of eight, in London; and was considered in great danger, but recovered.

"Case 2d.—Susannah was inoculated for the cow-pox, by Dr. Storey, at Penrith, when about a year old; and she had the small-pox in London, by inoculation, when she was about six. Her fever was very strong, and she had 25 small-pox.

"Case 3d.—William was vaccinated by Dr. Jenner at London, when he was eleven months old. He had been previously inoculated for the cow-pox, by Dr. Alcock of London, but did not take the infection. He had the small-pox by inoculation at the age of five years, when he had very strong fever, and twelve small-pox.

"The above children had the cow-pox regularly, and under the particular attention and approbation of Mr. Ballantine, a medical gentleman, their maternal grandfather, who considered them all free from any danger of infection.

"Case 4th.—Anne was inoculated thrice for the cow-pox, but did not take it—the fourth time by Dr. Jenner, who succeeded at the age of nine months. She was inoculated twelve months afterwards for the small-pox, without receiving the infection.

"Case 5th.—George was vaccinated at the age of five months, and took the infection. He was inoculated five months afterwards for the small-pox, which had no effect.

"Case 6th.—One of the servants who waited on these children children was vaccinated about twelve years since, and has not taken the small-pox."

The account of this family was handed to me by Mr. John Gough, of this neighbourhood, a gentleman well known to the philosophical world, who obtained it from the mother of the children, by inquiry.

On reviewing the whole of these cases, one cannot but incline to believe, that it is a difficult matter to discern that peculiar effect produced by the cow-pox on the human constitution, which renders it absolutely secure from the small-pox. That, in the majority of cases, this effect is produced, I would willingly hope: at the same time it is evident, that in the practice of the most experienced and attentive men, the cow-pock occasionally fails to furnish the constitution with any other power than that of moderating the violence of the small-pox: consequently, in those cases which go through the regular appearances of cow-pox, one cannot but suspect, that a criterion remains yet to be discovered by which we can be enabled to pronounce any patient to be absolutely secure from small-pox afterwards.

The cases furnished me by Mr. Redhead are the result of the inquiry and observations of a candid mind; and, with his leave, I have been induced, by a sense of duty, to lay them reluctantly before the profession, with no other design than that of recording (what appear to me) medical facts, for the purpose of exciting medical men to more minute observation in the practice of vaccination. That the cow-pock is a valuable discovery, no man can doubt; for, although it may fail to afford perfect security from the small-pox in many cases, yet it appears to have the power of mitigating the violence of that horrible disease in all: and, if it had no other effect, it ought to be considered as a blessing to the human race, and to be strenuously recommended by every one who has the welfare of mankind at heart.

In this town, vaccination has been generally practised during the last fifteen years; and, although the small-pox has been repeatedly in the town, yet no one case of small-pox after vaccination has occurred in the practice of any of the medical men, so far as they recollect. And, notwithstanding that the failures which have taken place at Ulverston, twenty-one miles from hence, produced considerable alarm, yet the inhabitants are so well convinced of the preventive power of the cow-pox, in the majority of cases, and of the mitigating power in all, that vaccination, I understand, goes on in that place as usual. Indeed, I hope that no man will ever meditate the cruelty to the human race of introducing or advocating the practice of variolous inoculation,

 unless unless it be to ascertain the effect of previous vaccination. For although, under strict management, variolous inoculation may be nearly as seldom fatal as vaccination, yet the great superiority of the latter over the former consists in its communicating a mild disease, which cannot be propagated but by direct or accidental inoculation; whilst the small-pox, either natural or inoculated, when introduced into towns, produces, by its contagious effluvium, most dreadful havoc amongst those prejudiced or unwary persons who may happen to be exposed to its influence.

*Kendal; Oct.* 29, 1816.

